# Web-based 3D-visualization of the DrugBank chemical space

**DOI:** 10.1186/s13321-016-0138-2

**Published:** 2016-05-04

**Authors:** Mahendra Awale, Jean-Louis Reymond

**Affiliations:** Department of Chemistry and Biochemistry, National Center of Competence in Research NCCR TransCure, University of Bern, Freiestrasse 3, 3012 Bern, Switzerland

**Keywords:** DrugBank, Chemical space, Visualization, Fingerprints, Molecular shape, Pharmacophores

## Abstract

**Background:**

Similarly to the periodic table for elements, chemical space offers an organizing principle for representing the diversity of organic molecules, usually in the form of multi-dimensional property spaces that are subjected to dimensionality reduction methods to obtain 3D-spaces or 2D-maps suitable for visual inspection. Unfortunately, tools to look at chemical space on the internet are currently very limited.

**Results:**

Herein we present webDrugCS, a web application freely available at www.gdb.unibe.ch to visualize DrugBank (www.drugbank.ca, containing over 6000 investigational and approved drugs) in five different property spaces. WebDrugCS displays 3D-clouds of color-coded grid points representing molecules, whose structural formula is displayed on mouse over with an option to link to the corresponding molecule page at the DrugBank website. The 3D-clouds are obtained by principal component analysis of high dimensional property spaces describing constitution and topology (42D molecular quantum numbers MQN), structural features (34D SMILES fingerprint SMIfp), molecular shape (20D atom pair fingerprint APfp), pharmacophores (55D atom category extended atom pair fingerprint Xfp) and substructures (1024D binary substructure fingerprint Sfp). User defined molecules can be uploaded as SMILES lists and displayed together with DrugBank. In contrast to 2D-maps where many compounds fold onto each other, these 3D-spaces have a comparable resolution to their parent high-dimensional chemical space.

**Conclusion:**

To the best of our knowledge webDrugCS is the first publicly available web tool for interactive visualization and exploration of the DrugBank chemical space in 3D. WebDrugCS works on computers, tablets and phones, and facilitates the visual exploration of DrugBank to rapidly learn about the structural diversity of small molecule drugs.

**Graphical abstract:**
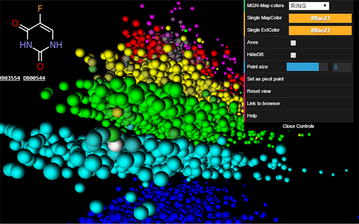
webDrugCS visualization of DrugBank projected in 3D MQN space color-coded by ring count, with pointer showing the drug 5-fluorouracil.

## Background

One of the defining features of organic chemistry is the extremely large diversity of possible molecules. The concept of chemical space, whereby molecules are annotated with a set of quantitative molecular properties and placed in a high-dimensional property space with each dimension corresponding to a different property, offers a practical approach to represent the structural diversity of large molecule collections [1–28]. Such high-dimensional spaces cannot be visualized directly but can be subjected to various dimensionality reduction methods to obtain 3D-spaces or 2D-maps suitable for visual inspection [29–32].

To make chemical space easier to inspect, we recently reported an interactive Java Applet representing databases of molecules as color-coded maps produced by projection of high-dimensional property spaces, defined by various molecular fingerprints, into two dimensions [32–37]. In these so-called Mapplets the computer screen shows a color-coded 2D-image where each pixel contains one or several molecules projected at that point. The average molecule contained in each pixel is displayed on a side-window on mouse over, with an option to open the complete list of molecules in the pixel in a secondary window, and subsequently to link selected molecules to the database entry, or to perform similarity searches in the parent high-dimensional property space. These Mapplets unfortunately suffer from the typical folding effects encountered when projecting high-dimensional property spaces into 2D [2, 6, 9, 28, 30, 32], which results in (a) many pixels containing molecules piled-up on top of each other, and (b) a poor correlation between distances on the 2D-map and distances in the original high-dimensional property space. In addition the Java Applets must be downloaded and run separately and are not platform independent.

Herein we report webDrugCS, a web application freely accessible at www.gdb.unibe.ch which addresses these limitations by enabling access to molecules via interactive color-coded 3D-spaces in a manner similar to the 2D-mapplet. The website visualizes DrugBank (http://www.drugbank.ca/), a public database listing over 6000 compounds currently in medical use either as FDA approved and marketed drug or as investigational drugs [38]. Similarly to our recently reported PDB-Explorer website to visualize the Protein Databank [39], webDrugCS uses the internet browser of the user to generate the display. DrugBank is represented in the form of color coded 3D-spaces obtained by principal component analysis (PCA) of five different multidimensional property spaces defined by five different fingerprints. These fingerprints describe constitution and topology (42D molecular quantum numbers, MQN), structural features (34D SMILES fingerprint SMIfp), molecular shape (20D atom pair fingerprint APfp), pharmacophores (55D atom category extended atom pair fingerprint Xfp) and substructures (1024D binary substructure fingerprint Sfp) (Table 1). The 3D-spaces are generated using three.js (http://threejs.org/), an open-source JavaScript library/API for animated 3D computer graphics in a web browser. Although less sophisticated than other chemical space visualization tools designed to assess compound collections [40–46], webDrugCS provides an unprecedented tool to look at DrugBank and rapidly learn about the structural diversity of small molecule drugs. This feature is not offered at the DrugBank website and at any other currently available online tools such as eDrug3D [47], SuperDrug [48], SuperPred [49], or BalestraWeb [50], which are primarily designed to address specific queries such as drug name, substructure, molecular formula or protein target by providing a limited number of answers.

**Table 1:** Fingerprints used in this study

Fingerprint	Feature perceived	Description	References
MQN	Composition	42D scalar fingerprint, counts 42 molecular quantum numbers (MQN) counting atom types, bond types, polar groups and topologies	[51, 52]
SMIfp	Composition	34D scalar fingerprint, counts 34 characters appearing in the SMILES notation of molecules	[35]
APfp	Shape	20D scalar fingerprint, each dimension counts the number of atom pairs at one particular topological distance between 1 and 20 bonds, normalized to HAC	[53]
Xfp	Pharmacophore	55D scalar fingerprint, category extended version of APfp counting the number of category atom pairs at one particular topological distance between 0 and 10 bonds, normalized to the number of category atoms, for categories: hydrophobic atoms, H-bond donor atoms, H-bond acceptor atoms, sp2 hybridized atoms, and HBA/HBD cross-pairs	[53]
Sfp	Substructure	1024D binary fingerprint, perceives the presence of substructures	[54]

## Results and discussion

### PCA of multidimensional property spaces

**Fig. 1:**
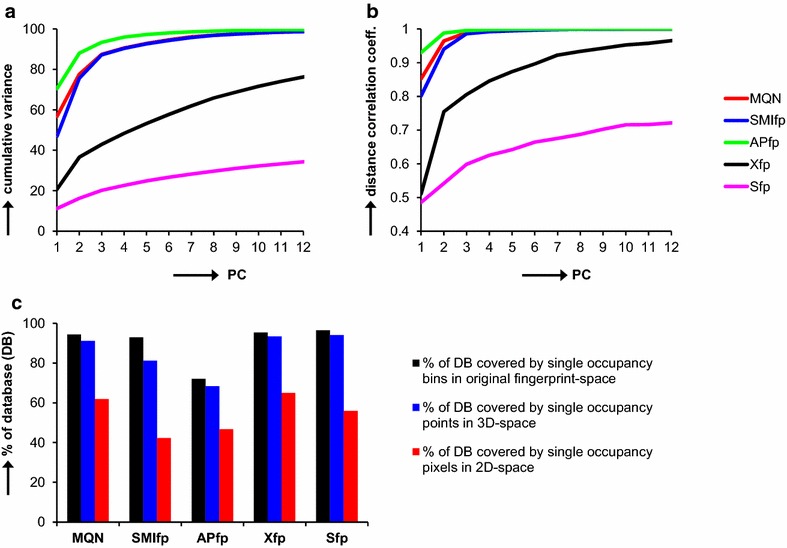
Analysis of the DrugBank database. **a** Percentage of data variance covered by increasing numbers of PC obtained by PCA of MQN-, SMIfp-, APfp-, Xfp- and Sfp-datasets of DrugBank. **b** Pearson’s correlation coefficient between pairwise euclidean distances in the n-dimensional PC-subspace and the respective original MQN, SMIfp, APfp, Xfp and Sfp fingerprint spaces, calculated from analyzing 36 M molecule pairs in the DrugBank database. **c** Percentage of the DrugBank database considering all single occupied bins in the original fingerprint space (*black*), grid points in 3D-space (*blue*) and pixels in 2D-space (*red*). A bin is defined as one particular fingerprint value combination. The 3D-spaces were generated by projecting DrugBank onto a grid of 300 × 300 × 300 grid points. The 2D-maps were generated by projecting the DrugBank onto a map of 300 × 300 pixels

In a multidimensional property space dimensions and the position (coordinates) of any molecule are defined by a set of molecular descriptors. PCA is performed as a dimensionality reduction method to obtain 3D- or 2D-representations. In these projections the position of any molecule is defined by its coordinates in the first three respectively two principal components (PCs). Here PCA is used to project DrugBank from each of the five property spaces defined by the fingerprints MQN, SMIfp, APfp, Xfp and Sfp onto the corresponding 3D-space or 2D-map. The cumulative coverage of data variance within the first 3 PCs is larger than 75 % in the case of the fingerprints MQN, SMIfp and APfp, which are relatively simple descriptions of the molecules resulting in a relatively low number of dimensions (Fig. 1a). In these cases a very good correlation is observed between distances in the original high-dimensional property space and the 3D-projection (Fig. 1b). The situation is less optimal for the more complex and higher dimensional fingerprints Xfp and Sfp, where only 42 % respectively 20 % of data variance is covered within the first three PCs. Nevertheless the correlation between distances in the original property space and the 3D-space resulting from PCA is still acceptable (Xfp: 0.8, Sfp: 0.6), implying that these 3D-spaces still contain relevant information about the position of molecules in the original high-dimensional Xfp and Sfp spaces. In particular nearest neighbours in each of the 3D spaces are for the most part closely related molecules in the corresponding high-dimensional property space.

One of the remarkable aspects of the 3D-spaces concerns the resolution of compounds into individual 3D-grid positions after assigning molecules to a 3D-grid point in a 300 × 300 × 300 box covering the range of (PC1, PC2, PC3) values. In the original multidimensional property spaces an excellent resolution is obtained for DrugBank in the sense that almost all DrugBank molecules are encoded by a unique fingerprint bit value combination. This resolution is largely preserved upon PCA and assignment to the 3D-grid, as can be judged by the fact that the percentage of molecules appearing in singly occupied 3D-grid points is comparable to the percentage of molecule having a single fingerprint bit-value combination. The 3D-space is clearly superior in that matter to the 2D-map, where compounds are assigned to 2D-pixels in a 300 × 300 square covering the range of (PC1, PC2) values. In this case a significant folding occurs and only 40–60 % of the compounds appear in single occupied 2D-pixels (Fig. 1c).

**Fig. 2:**
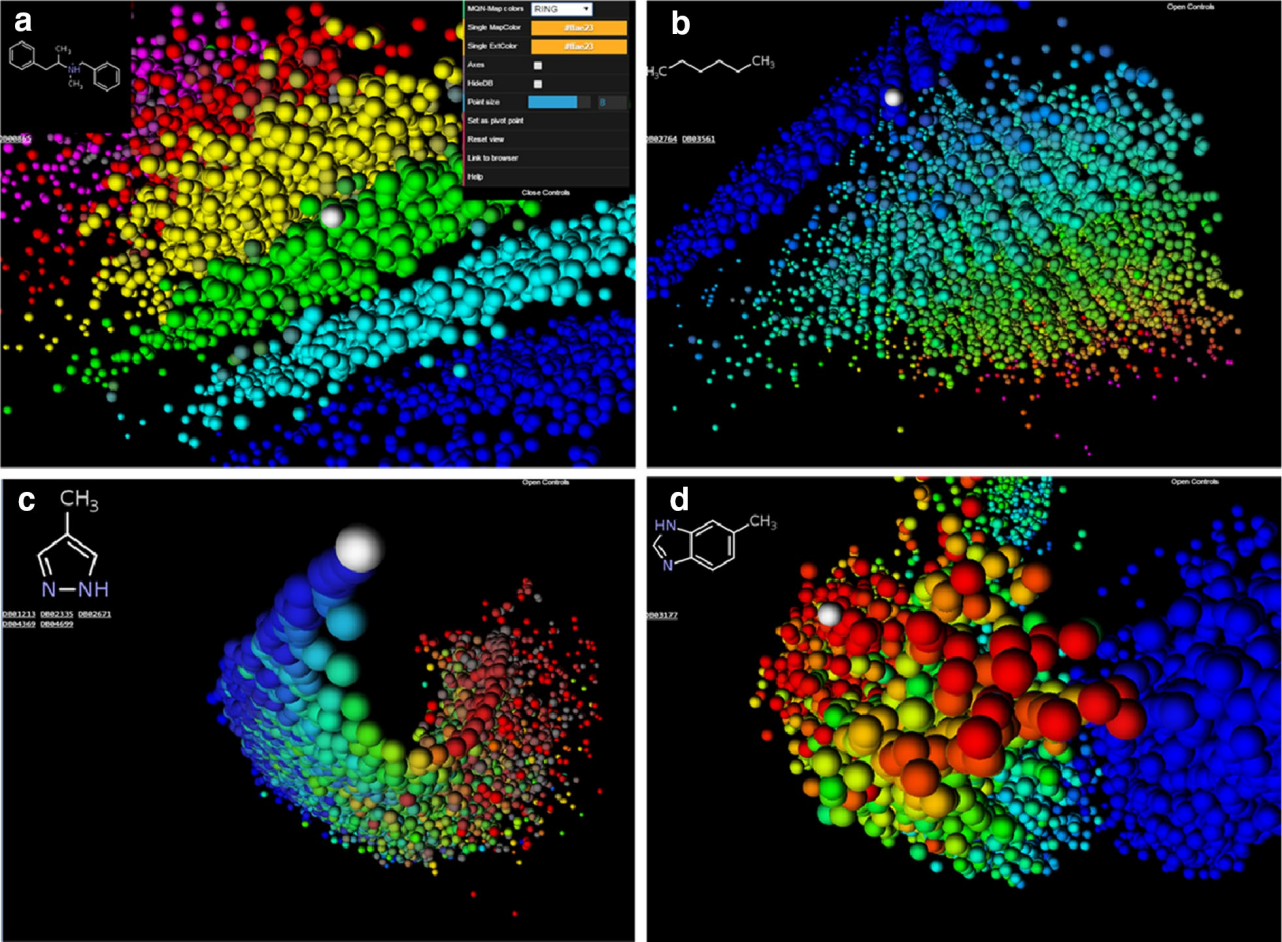
Color coded 3D-spaces of the DrugBank chemical space obtained by taking snapshots from the webDrugCS website (www.gdb.unibe.ch). The color changes in the range blue → cyan → green → yellow → red → magenta with increasing property value. **a** MQN 3D-space color coded by ring count, shown with open control panel **b** SMIfp 3D-space color coded by the number of aromatic carbon atoms. **c** APfp 3D-space color coded by rotatable bond count. **d** Sfp 3D-space color coded by the fraction of aromatic atoms. The molecule shown in the viewer window is located in the mouse over pixel, which is marked as a *white sphere* in the image

As an additional noticeable feature the 3D-representations of the various property spaces represent DrugBank an intuitively logical spatial organization which can be visualized by color-coding each grid-point with a selected property value. As illustrated by screen-shots taken from the web application webDrugCS (details discussed below), striking features include for example parallel stripes grouping compounds of increasing ring count in the MQN 3D-space (Fig. 2a), the separation of molecules according to their number of aromatic carbon atoms in the SMIfp 3D-space (Fig. 2b) and according to their rotatable bond count in the APfp 3D-space (Fig. 2c), and the global separation of the Sfp 3D-space according to the fraction of aromatic atoms (Fig. 2d).

### webDrugCS

**Fig. 3:**
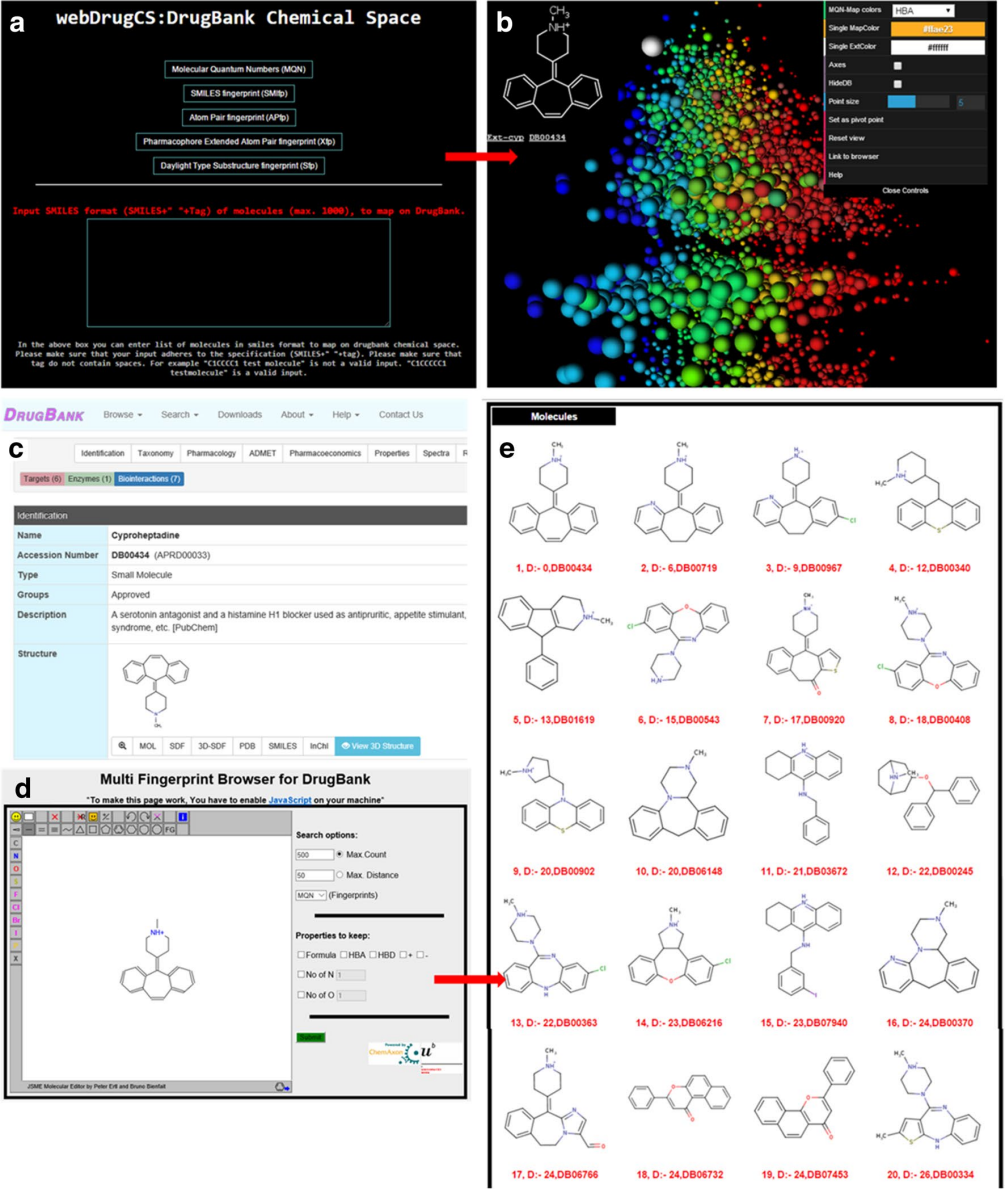
The webDrugCS website and its functionalities. **a** Starting page of the webDrugCS. MQN, SMIfp, APfp, Xfp and Sfp 3D-spaces of the DrugBank database can be accessed by clicking on respective buttons. A list of molecules to be mapped on any 3D-space of the DrugBank database can be entered (format: SMILES) into the text box provided in the lower part of the page. See main text for the exact input format and details. **b** Interactive visualization window for MQN 3D-spaces obtained by clicking the button corresponding to MQN in the starting page. The 3D-space is shown with color coding using HBA atom count. On mouse over the panel at *top left* displays the molecule at the corresponding grid point. The example shown is cyproheptadine. **c** The DrugBank page for cyproheptadine was obtained by clicking the drug code displayed in the *left panel* in **b**. **d** Multifingerprint browser window for DrugBank with the cyproheptadine as query, obtained by clicking the “Link to browser” option in the control panel (*top right panel* in **b**). **e** Results window displaying the MQN-nearest neighbors of the query cyproheptadine in DrugBank

WebDrugCS (www.gdb.unibe.ch) is an online application for interactive visualization and exploration of DrugBank in color coded 3D property spaces. The application works on computers, tablets and phones. The starting page of webDrugCS (Fig. 3a) provides two options (1) *Selection of molecular fingerprint:* Choose between MQN, SMIfp, APfp, Xfp and Sfp fingerprint 3D-spaces by clicking the corresponding field, which opens a new browser tab. (2) *External chemical library:* The user can input up to 1000 additional molecules in SMILES format, which will be displayed together with DrugBank in any of the selected 3D-spaces. Each of the lines in the text box must represent an individual molecule as SMILES followed by a space and its name or tag. External molecules are viewed by default as dark violet colored grid points.

The graphical user interface (GUI) of the interactive visualization window is exemplified here with the MQN 3D-space. The GUI consists of a main panel, a molecule view panel, and a control panel. The main panel occupies the entire screen area and displays the 3D-space (Fig. 3b). Each point in the 3D-space is represented as sphere, whose size depends on its distance to the camera. The view angle rotates by dragging the mouse upon left click, and the wheel controls the zoom in/out function.

The view panel is positioned at upper left and shows the structural formula and DrugBank ID of the molecule at the current mouse-over 3D-grid point. Upon selecting a grid point by double click, one can then link to the molecule page at the DrugBank webpage by clicking on the DrugBank ID displayed below the structural formula (Fig. 3c), or access a similarity browser to search for nearest neighbours in the original high-dimensional fingerprint space via the control panel (Fig. 3d/e).

The control panel at top right lists options to change the 3D-space view. Lines 1–3: select a color code according to a descriptor, or a single color code for DrugBank and the uploaded molecule list. Line 4: display the reference 3D-axes. Line 5: hide the DrugBank grid points, leaving only the molecules uploaded by the user as visible points. Line 6: change the 3D-grid point sphere size. Line 7: set the currently selected 3D-grid point as reference pivot point for the 3D-space (after selecting a grid point by double click). Line 8: Reset the view to the default entry view. Line 9: Link to the fingerprint similarity browser, which opens as an additional tab. This browser allows one to perform nearest neighbour searches in DrugBank in any of the five original high-dimensional fingerprint spaces. The browser is built in the same manner as our recently reported ChEMBL similarity browser [32]. Line 10: help function listing the different options.

**Fig. 4:**
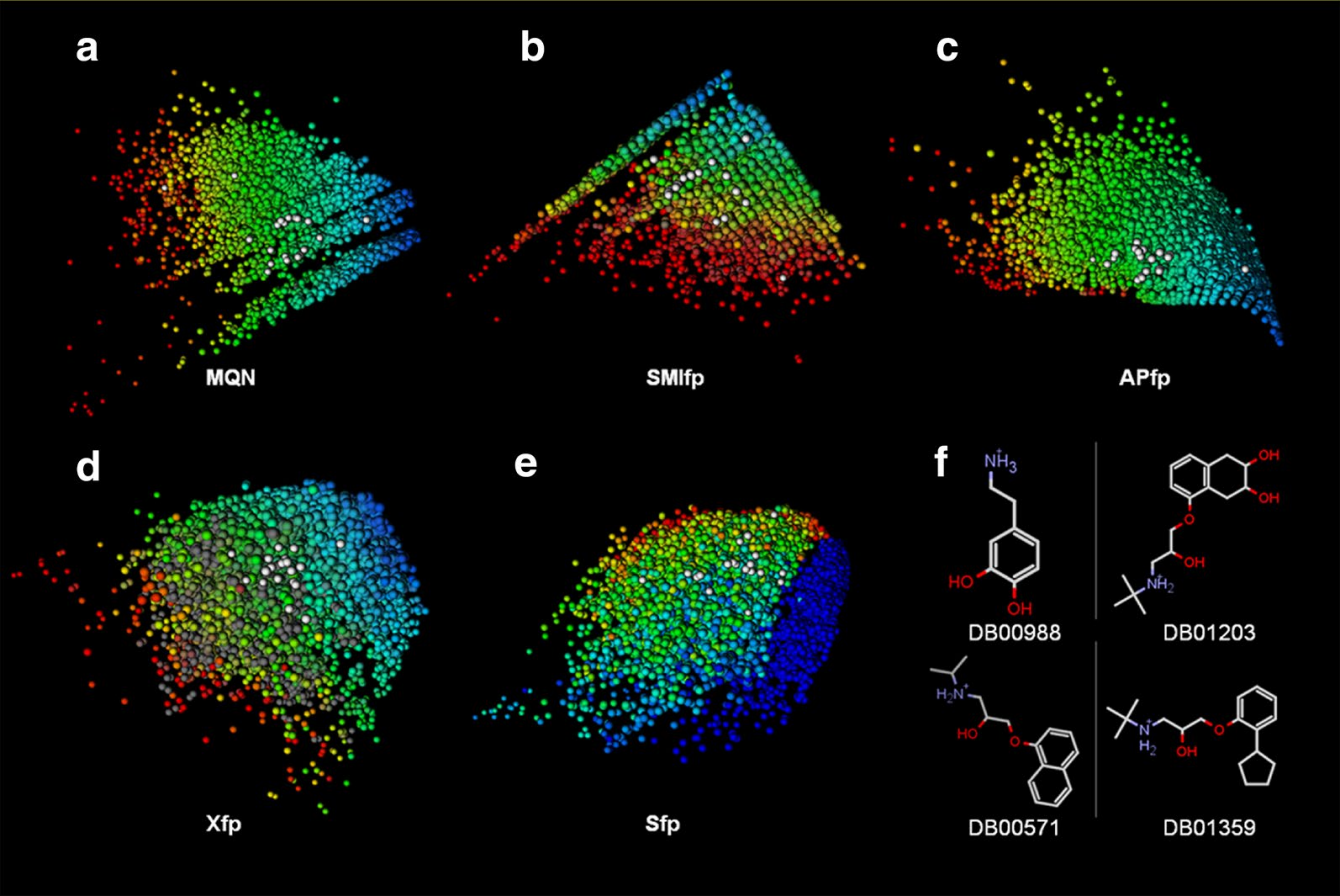
Mapping of 20 DrugBank comopunds annotated with β1-adrenergic receptor antagonist activity in ChEMBL using the extrenal library option in webDrugCS. The 20 extrenal compounds are shown as *white dots*, overlayed on DrugBank shown in color-coded representation. Heavy atom count color coding was used for the MQN map (**a**), SMIfp map (**b**), APfp map (**c**) and Xfp map (**d**), while the N–C=C substructure cound is used for Sfp (**e**). Four of the 20 selected drugs are shown in **e**

The external chemical library option in the entry panel (Fig. 3a) is illustrated here for mapping 24 drugs from DrugBank annotated in ChEMBL as β1-adrenergic receptor antagonists. These typical drugs contain a short aliphatic amine or aminoalcohol connected to a mono- or bicyclic aromatic nucleus. Due to their comparable overall composition, molecular shape, pharmacophore and substructural elements these 20 drugs form a relatively tight group in each of the five property spaces in webDrugCS (Fig. 4). In general series of structurally related molecules appear grouped in the various 3D-spaces available with webDrugCS. Note that the option “hideDB” in the control panel allows one to remove the drugbank compounds, which leaves only the external library as visible points.

## Conclusion

webDrugCS represents the first online application for visualizing DrugBank in five different 3D property spaces on computers, tablets or phones. In contrast to the other database exploration tools, webDrugCS can be used for curiosity driven exploration independently of specific queries, and is particularly suitable to rapidly gain an overview of the structures of drug molecules. While the present web-based application is currently limited to displaying of a few thousand points, the method might be applicable to displaying larger databases of millions of molecules if significant coding progress can be made.

## Methods

*Databases* The DrugBank database was downloaded in SDF format from http://www.drugbank.ca/. Molecules were processed by checking for valency error, removing counter ions and adjusting their ionization state to pH 7.4, using an in-house built java program utilizing Java Chemistry library (JChem) from ChemAxon, Pvt. Ltd., as a starting point. Duplicates and molecules larger than 50 heavy atoms were removed from the database.

*Fingerprints* Calculation of MQN, SMIfp, APfp and Xfp fingerprints are discussed in detail in the respective publications from our group. Fingerprints were calculated as described previously using plugins provided in JChem chemistry library.

### Principal component analysis

The PCA for each database was performed using an in house written Java program utilizing some of the available mathematical functions from JSci (A science API for Java: http://jsci.sourceforge.net/). The Java source code is based on the tutorial of Lindsay I. Smith (http://www.cs.otago.ac.nz/cosc453/student_tutorials/principal_components.pdf).

### 3D-space and color coding

The PC-1, PC-2 and PC-3 values were calculated for each molecule in the database. The largest (PCmax) and smallest (PCmin) PC values appearing in the PC-1 or PC-2 or PC-3 values were used to define the value range ΔPC = PCmax − PCmin and set the binning scale as ΔPC/300. The PC-1, PC-2 and PC-3 values were binned onto 300 × 300 × 300 3D-grids using the same absolute bin size on the PC-1, PC-2 and PC-3 axis. Each molecule was assigned to a point on this 3D-grid. The Hue–Saturation–Lightness (HSL) color space was used for color coding, setting the hue value according to the average value of the selected molecular property across all molecules residing at that grid point, and the saturation according to the standard deviation of that value across all molecules within ±5 grid points in each direction. As a result the color change blue–cyan–green–yellow–red–magenta shows an increasing average value of property in a grid point, and saturation to grey indicates a strong gradient of the value in the vicinity.

### webDrugCS

The core part of webDrugCS for 3D-rendering and visualization is supported by the Three.js (http://threejs.org/), an open-source JavaScript library/API to create and display animated 3D computer graphics in a web browser. Three.js uses WebGL and runs across various browsers without need for any additional plugins. The webDrugCS has been successfully tested on IE, Chrome and Opera browsers. The only requirement for the webDrugCS is to have JavaScript enabled in a web browser. The source code of the webDrugCS visualizer is available for download at https://github.com/mahendra-awale/webDrugCS.

## References

[1] Pearlman RS, Smith KM (1998). Novel software tools for chemical diversity. Persp Drug Discov Des.

[2] Oprea TI, Gottfries J (2001). Chemography: the art of navigating in chemical space. J Comb Chem.

[3] Takahashi Y, Konji M, Fujishima S (2003). MolSpace: a computer desktop tool for visualization of massive molecular data. J Mol Graph Model.

[4] Haggarty SJ, Clemons PF, Wong JC, Wong JF, Schreiber SL (2004). Mapping chemical space using molecular descriptors and chemical genetics: deacetylase inhibitors. Comb Chem High Throughput Screen.

[5] Eckert H, Bajorath J (2007). Molecular similarity analysis in virtual screening: foundations, limitations and novel approaches. Drug Discov Today.

[6] Medina-Franco JL, Maggiora GM, Giulianotti MA, Pinilla C, Houghten RA (2007). A Similarity-based data-fusion approach to the visual characterization and comparison of compound databases. Chem Biol Drug Des.

[7] Medina-Franco JL, Martinez-Mayorga K, Giulianotti MA, Houghten RA, Pinilla C (2008). Visualization of the chemical space in drug discovery. Curr Comput-Aided Drug Des.

[8] Medina-Franco JL, Martinez-Mayorga K, Bender A, Marin RM, Giulianotti MA, Pinilla C, Houghten RA (2009). Characterization of activity landscapes using 2D and 3D similarity methods: consensus activity cliffs. J Chem Inf Model.

[9] Rosen J, Gottfries J, Muresan S, Backlund A, Oprea TI (2009). Novel chemical space exploration via natural products. J Med Chem.

[10] Singh N, Guha R, Giulianotti MA, Pinilla C, Houghten RA, Medina-Franco JL (2009). Chemoinformatic analysis of combinatorial libraries, drugs, natural products, and molecular libraries small molecule repository. J Chem Inf Model.

[11] Ivanenkov YA, Savchuk NP, Ekins S, Balakin KV (2009). Computational mapping tools for drug discovery. Drug Discov Today.

[12] Akella LB, DeCaprio D (2010). Cheminformatics approaches to analyze diversity in compound screening libraries. Curr Opin Chem Biol.

[13] Geppert H, Vogt M, Bajorath J (2010). Current trends in ligand-based virtual screening: molecular representations, data mining methods, new application areas, and performance evaluation. J Chem Inf Model.

[14] Reymond JL, Van Deursen R, Blum LC, Ruddigkeit L (2010). Chemical space as a source for new drugs. MedChemComm.

[15] Le Guilloux V, Colliandre L, Bourg S, Guénegou G, Dubois-Chevalier J, Morin-Allory L (2011). Visual characterization and diversity quantification of chemical libraries: 1. Creation of delimited reference chemical subspaces. J Chem Inf Model.

[16] Reutlinger M, Guba W, Martin RE, Alanine AI, Hoffmann T, Klenner A, Hiss JA, Schneider P, Schneider G (2011). Neighborhood-preserving visualization of adaptive structure-activity landscapes: application to drug discovery. Angew Chem Int Ed Engl.

[17] Owen JR, Nabney IT, Medina-Franco JL, López-Vallejo F (2011). Visualization of molecular fingerprints. J Chem Inf Model.

[18] Medina-Franco JL, Yongye AB, Pérez-Villanueva J, Houghten RA, Martínez-Mayorga K (2011). Multitarget structure–activity relationships characterized by activity–difference maps and consensus similarity measure. J Chem Inf Model.

[19] Maggiora GM, Shanmugasundaram V (2011). Molecular similarity measures. Methods Mol Biol (Clifton NJ).

[20] Yoo J, Medina-Franco J (2011). Chemoinformatic approaches for inhibitors of DNA methyltransferases: comprehensive characterization of screening libraries. Comput Mol Biosci.

[21] Gutlein M, Karwath A, Kramer S: CheS-Mapper—chemical space mapping and visualization in 3D. J Cheminform. 2012; 4:Article 7. http://www.jcheminf.com/content/4/1/7. Accessed 14 July 2015 10.1186/1758-2946-4-7 PMC3331825 22424447

[22] Ertl P, Rohde B: The molecule cloud—compact visualization of large collections of molecules. J Cheminform. 2012; 4:Article 12. http://www.jcheminf.com/content/14/11/12. Accessed 16 Dec 2012 10.1186/1758-2946-4-12 PMC3403880 22769057

[23] Lachance H, Wetzel S, Kumar K, Waldmann H (2012). Charting, navigating, and populating natural product chemical space for drug discovery. J Med Chem.

[24] Medina-Franco JL, Aguayo-Ortiz R (2013). Progress in the visualization and mining of chemical and target spaces. Mol Inf.

[25] Hoksza D, Skoda P, Vorsilak M, Svozil D: Molpher: a software framework for systematic chemical space exploration. J Cheminform. 2014; 6:Article 7. http://www.jcheminf.com/content/6/1/7. Accessed 14 July 2015 10.1186/1758-2946-6-7 PMC3998053 24655571

[26] Miyao T, Reker D, Schneider P, Funatsu K, Schneider G (2015). Chemography of natural product space. Planta Med.

[27] Rodrigues T, Hauser N, Reker D, Reutlinger M, Wunderlin T, Hamon J, Koch G, Schneider G (2015). Multidimensional de novo design reveals 5-HT2B receptor-selective ligands. Angew Chem Int Ed Engl.

[28] Sander T, Freyss J, von Korff M, Rufener C (2015). Datawarrior: an open-source program for chemistry aware data visualization and analysis. J Chem Inf Model.

[29] Digles D, Ecker GF (2011). Self-organizing maps for in silico screening and data visualization. Mol Inf.

[30] Gaspar HA, Baskin II, Marcou G, Horvath D, Varnek A (2014). Chemical data visualization and analysis with incremental generative topographic mapping: big data challenge. J Chem Inf Model.

[31] Deng Z-L, Du C-X, Li X, Hu B, Kuang Z-K, Wang R, Feng S-Y, Zhang H-Y, Kong D-X (2013). Exploring the biologically relevant chemical space for drug discovery. J Chem Inf Model.

[32] Awale M, Reymond JL (2015). Similarity mapplet: interactive visualization of the directory of useful decoys and ChEMBL in high dimensional chemical spaces. J Chem Inf Model.

[33] Awale M, Reymond JL (2012). Cluster analysis of the DrugBank chemical space using molecular quantum numbers. Bioorg Med Chem.

[34] Awale M, van Deursen R, Reymond JL (2013). MQN-Mapplet: visualization of chemical space with interactive maps of DrugBank, ChEMBL, PubChem, GDB-11, and GDB-13. J Chem Inf Model.

[35] Schwartz J, Awale M, Reymond JL (2013). SMIfp (SMILES fingerprint) chemical space for virtual screening and visualization of large databases of organic molecules. J Chem Inf Model.

[36] Ruddigkeit L, Awale M, Reymond JL (2014). Expanding the fragrance chemical space for virtual screening. J Cheminform.

[37] Reymond JL (2015). The chemical space project. Acc Chem Res.

[38] Knox C, Law V, Jewison T, Liu P, Ly S, Frolkis A, Pon A, Banco K, Mak C, Neveu V (2011). DrugBank 3.0: a comprehensive resource for ‘Omics’ research on drugs. Nucleic Acids Res.

[39] Jin X, Awale M, Zasso M, Kostro D, Patiny L, Reymond JL (2015). PDB-Explorer: a web-based interactive map of the protein data bank in shape space. BMC Bioinformatics.

[40] Gutlein M, Karwath A, Kramer S (2012). CheS-Mapper—chemical space mapping and visualization in 3D. J Cheminf.

[41] Wetzel S, Klein K, Renner S, Rauh D, Oprea TI, Mutzel P, Waldmann H (2009). Interactive exploration of chemical space with Scaffold Hunter. Nat Chem Biol.

[42] Ertl P, Rohde B (2012). The molecule cloud—compact visualization of large collections of molecules. J Cheminf.

[43] Hoksza D, Skoda P, Vorsilak M, Svozil D (2014). Molpher: a software framework for systematic chemical space exploration. J Cheminf.

[44] Hilbig M, Rarey M (2015). MONA 2: a light cheminformatics platform for interactive compound library processing. J Chem Inf Model.

[45] Lewis R, Guha R, Korcsmaros T, Bender A (2015). Synergy Maps: exploring compound combinations using network-based visualization. J Cheminf.

[46] Korb O, Kuhn B, Hert J, Taylor N, Cole J, Groom C, Stahl M (2016). Interactive and versatile navigation of structural databases. J Med Chem..

[47] Lewell XQ, Jones AC, Bruce CL, Harper G, Jones MM, McLay IM, Bradshaw J (2003). Drug rings database with web interface. A tool for identifying alternative chemical rings in lead discovery programs. J Med Chem.

[48] Goede A, Dunkel M, Mester N, Frommel C, Preissner R (2005). SuperDrug: a conformational drug database. Bioinformatics.

[49] Nickel J, Gohlke B-O, Erehman J, Banerjee P, Rong WW, Goede A, Dunkel M, Preissner R (2014). SuperPred: update on drug classification and target prediction. Nucleic Acids Res.

[50] Cobanoglu MC, Oltvai ZN, Taylor DL, Bahar I (2015). BalestraWeb: efficient online evaluation of drug–target interactions. Bioinformatics.

[51] Nguyen KT, Blum LC, van Deursen R, Reymond J-L (2009). Classification of organic molecules by molecular quantum numbers. ChemMedChem.

[52] van Deursen R, Blum LC, Reymond JL (2010). A searchable map of PubChem. J Chem Inf Model.

[53] Awale M, Reymond JL (2014). Atom pair 2D-fingerprints perceive 3D-molecular shape and pharmacophores for very fast virtual screening of ZINC and GDB-17. J Chem Inf Model.

[54] Hagadone TR (1992). Molecular substructure similarity searching: efficient retrieval in two-dimensional structure databases. J Chem Inf Comput Sci.

